# Supplementary material for: On the Gaussian distribution of the Mann-Kendall tau in the case of autocorrelated data
Source: PLoS One. 2026 Feb 19;21(2):e0333224. doi: 10.1371/journal.pone.0333224 (PMC12919832; doi:10.1371/journal.pone.0333224)
Supplement: S1 Appendix — (PDF) [file pone.0333224.s001.pdf]

S1 Appendix

1 Proofs

This appendix contains the proofs of the main results presented in the paper. To this end, we begin by establishing a number of auxiliary lemmas which will serve as intermediate steps for the proofs of the main results.

1.1 Other Lemmas

Lemma A1 (Positivity). *Let* $0 \leq x \leq y \leq 1$ *and* $0 < \alpha < 1$:

$$\sqrt{1-\alpha^{1-y+x}}(\alpha^{1-y} - \alpha - \alpha^{y-x} + \alpha^{x}) + \sqrt{1-\alpha^{y}}(-\alpha^{1-y} - \alpha + \alpha^{y-x} + \alpha^{x}) \geq 0 \quad \text{(A1)}$$

Proof of Lemma A1. We write it such that both terms are positive

$$\begin{aligned}
&(\sqrt{1-\alpha^{1-y+x}} + \sqrt{1-\alpha^{y}})(\alpha^{x} - \alpha) \geq (\sqrt{1-\alpha^{1-y+x}} - \sqrt{1-\alpha^{y}})(\alpha^{y-x} - \alpha^{1-y}) \\
\iff &|1-\alpha^{1-y+x} - (1-\alpha^{y})|(\alpha^{x} - \alpha) \geq (\sqrt{1-\alpha^{1-y+x}} - \sqrt{1-\alpha^{y}})^2 |\alpha^{y-x} - \alpha^{1-y}| \\
\iff &\alpha^{x}|\alpha^{1-y} - \alpha^{y-x}|(\alpha^{x} - \alpha) \geq (\sqrt{1-\alpha^{1-y+x}} - \sqrt{1-\alpha^{y}})^2 |\alpha^{y-x} - \alpha^{1-y}| \\
\iff &\alpha^{x}(\alpha^{x} - \alpha) \geq (\sqrt{1-\alpha^{1-y+x}} - \sqrt{1-\alpha^{y}})^2
\end{aligned}$$

Let $f(y) = (\sqrt{1-\alpha^{1-y+x}} - \sqrt{1-\alpha^{y}})^2$

$$\begin{aligned}
\max_{x \leq y \leq 1} f(y) &\leq (\max_{x \leq y \leq 1}(\sqrt{1-\alpha^{1-y+x}}) - \min_{x \leq y \leq 1}(\sqrt{1-\alpha^{y}}))^2 \\
&= (\min_{x \leq y \leq 1}(\sqrt{1-\alpha^{1-y+x}}) - \max_{x \leq y \leq 1}(\sqrt{1-\alpha^{y}}))^2 \\
&= (\sqrt{1-\alpha} - \sqrt{1-\alpha^{x}})^2 \\
&= f(x)
\end{aligned}$$

We then only need to show that:

$$\alpha^{x}(\alpha^{x} - \alpha) \geq (\sqrt{1-\alpha} - \sqrt{1-\alpha^{x}})^2, \quad 0 \leq x \leq 1$$

It is equivalent to:

$$\begin{aligned}
&x(x-\alpha) \geq (\sqrt{1-\alpha} - \sqrt{1-x})^2, \quad \alpha \leq x \leq 1 \\
\iff &x(x-(1-y)) \geq (\sqrt{y} - \sqrt{1-x})^2, \quad 1-y \leq x \leq 1
\end{aligned}$$

Let $g(x) = x(x-(1-y)) - (\sqrt{y} - \sqrt{1-x})^2$. We get $g(1-y) = g(1) = 0$.

$$\begin{aligned}
g'(x) &= 2x - (1-y) - 2(\sqrt{y} - \sqrt{1-x})(\frac{1}{2\sqrt{1-x}}) \\
g'(x) &= 2x + y - \frac{\sqrt{y}}{\sqrt{1-x}}
\end{aligned}$$

$g'(1-y) = 1-y = \alpha > 0$ and $g'(1^-) = -\infty$. We only need to show that $g'(x)$ cancels only once over $[1-y, 1]$.

$$\begin{aligned} g'(x) = 0 &\iff 2x + y = \frac{\sqrt{y}}{\sqrt{1-x}} \\ &\implies (2x+y)^2 = \frac{y}{1-x} \end{aligned}$$

Defining P as $P(x) := (1-x)(2x+y)^2 - y$, it is a third degree polynomial with $P(-\infty) = +\infty, P(-y/2) < 0, P(1-y) = y((2-y)^2 - 1) > 0$ and $P(1) < 0$. Then P has only one root with $1-y < x < 1$, then $g'(x)$ cancels only once over $]1-y, 1[$. The result is therefore proved. □

Lemma A2. *Let* $0 \leq a, b, c \leq 1$*, then:*

$$arcsin(a) + arcsin(b) + arcsin(c) = \frac{\pi}{2} \Leftrightarrow a^2 + b^2 + c^2 + 2abc = 1 \tag{A2}$$

Proof of Lemma A2. We have $\sin(\arcsin(a)) = a, \cos(\arcsin(a)) = \sqrt{1-a^2}$. By taking the sinus,

$$\begin{aligned} arcsin(a) = \frac{\pi}{2} - arcsin(b) - arcsin(c) &\Leftrightarrow a = \sqrt{1-b^2}\sqrt{1-c^2} - bc \\ &\Leftrightarrow (a+bc)^2 = (1-b^2)(1-c^2), \quad (0 \leq a, b, c \leq 1) \\ &\Leftrightarrow a^2 + b^2 + c^2 + 2abc = 1 \end{aligned}$$

□

Lemma A3. *Let* $n \geq 3, 0 < j < k < n$ *be integers and* $L^n(j,k) = \arcsin\left(\frac{k-j}{\sqrt{k}\sqrt{n-j}}\right)$. *Then,*

$$L^n(j,k) + L^n(j, n-k+j) + L^n(n-k, n-k+j) = \frac{\pi}{2} \tag{A3}$$

Proof of Lemma A3. Let $0 < j < k < n$, and $a = \frac{k-j}{\sqrt{k}\sqrt{n-j}}, b = \frac{n-k}{\sqrt{n-k+j}\sqrt{n-j}}, c = \frac{j}{\sqrt{n-k+j}\sqrt{k}}$, we have $0 \leq a, b, c \leq 1$ and:

$$\begin{aligned} \text{(A3)} &\Leftrightarrow a^2 + b^2 + c^2 + 2abc = 1, \quad \text{(Lemma } A2) \\ &\Leftrightarrow \frac{(k-j)^2}{k(n-j)} + \frac{(n-k)^2}{(n-j)(n-k+j)} + \frac{j^2}{k(n-k+j)} + 2\frac{j(k-j)(n-k)}{k(n-j)(n-k+j)} = 1 \\ &\Leftrightarrow (k-j)^2(n-k+j) + (n-k)^2 k + j^2(n-j) + 2j(k-j)(n-k) = k(n-j)(n-k+j) \\ &\Leftrightarrow n^2k - nk^2 - j^2k + jk^2 = k(n-j)(n-k+j) \end{aligned}$$

Developing the last equation we get the result. □

1.2 Proofs of the main text

Proof - Lemma 1. We define $\tilde{r}(i,j,k,l) = \text{corr}\left(X_j - X_i, X_l - X_k\right)$.

Let $1 \leq i < j, 1 \leq k < l, \tilde{r}(i,j,k,l) := \frac{\tilde{\rho}(|l-j|) - \tilde{\rho}(|l-i|) - \tilde{\rho}(|k-j|) + \tilde{\rho}(|k-i|)}{2\sqrt{1-\tilde{\rho}(|j-i|)}\sqrt{1-\tilde{\rho}(|l-k|)}}$. Using equations (3) and (7), we get:

$$
\begin{aligned}
\mathbb{V}(\tau_n) &= \frac{2}{\pi\binom{n}{2}^2}\sum_{1\le i<j\le n}\sum_{1\le k<l\le n}\arcsin(\tilde{r}(i,j,k,l))\\
&= \frac{4}{\pi\binom{n}{2}^2}\left(\sum_{1\le i<j<k<l\le n}\arcsin(\tilde{r}(i,j,k,l)) + \sum_{1\le i<k<j<l\le n}\arcsin(\tilde{r}(i,j,k,l))\right.\\
&+ \left.\sum_{1\le i<k<l<j\le n}\arcsin(\tilde{r}(i,j,k,l))\right) + o(1)\\
&= \frac{16}{\pi n^4}\sum_{1\le i<j<k<l\le n}\big(\arcsin(\tilde{r}(i,j,k,l)) + \arcsin(\tilde{r}(i,k,j,l)) + \arcsin(\tilde{r}(i,l,j,k))\big) + o(1)
\end{aligned}
$$

Because $\tilde{r}(i,j,k,l) = \tilde{r}(0, j-i, k-i, l-i), \forall i<j, k<l$,

$$
\sum_{1\le i<j<k<l\le n}\arcsin(\tilde{r}(i,j,k,l)) = \sum_{l=3}^{n}(n+1-l)\sum_{0<j<k<l}\arcsin(\tilde{r}(0,j,k,l)). \tag{A4}
$$

Finally, we get for one sum:

$$
\begin{aligned}
\lim_{n\to\infty}\frac{16}{\pi n^4}\sum_{1\le i<j<k<l\le n}\arcsin(\tilde{r}(i,j,k,l)) &= \lim_{n\to\infty}\frac{16}{\pi n^4}n\sum_{l=3}^{n}(1-\frac{l}{n})\sum_{0<j<k<l}\arcsin(\tilde{r}(0,\frac{j}{n},\frac{k}{n},\frac{l}{n}))\\
&= \frac{16}{\pi}\int_0^1(1-z)\int_0^z\int_0^y\arcsin(r(0,x,y,z))\mathrm{d}x\mathrm{d}y\mathrm{d}z
\end{aligned}
$$

Where the last equality is by definition of a Riemann sum. By doing the same for the two other sums, we get the result.

□

Proof - Lemma 2. Let $X_{t+1} = kX_t + \epsilon_{t+1}, \quad \epsilon_t \sim \mathcal{N}(0, 1-k^2), 0\le k<1, 1\le t\le q$

Let $Y_t = \sum_{i=t}^{q+t-1} X_i$.

$$
Y_{t+1} = \sum_{i=t+1}^{q+t} X_i = \sum_{i=t}^{q+t-1}(kX_i + \epsilon_{i+1})
$$

$$
Y_{t+1} = kY_t + \sum_{i=t+1}^{q+t}\epsilon_i
$$

As the (ϵ_j) are independent and identically distributed, one can do a change of variable and thus Y follows an ARMA(1,q-1).

$$
\begin{aligned}
\mathbb{V}(Y) &= \sum_{i=1}^{q}\sum_{j=1}^{q}\mathbb{E}(X_iX_j)\\
&= \sum_{i=1}^{q}\left(\sum_{j=1}^{i}k^{i-j} + \sum_{j=i}^{q}k^{j-i} - 1\right)\\
&= \sum_{i=1}^{q}\left(\frac{1-k^i}{1-k} + \sum_{j=i}^{q}\frac{1-k^{q-i+1}}{1-k}\right) - q\\
&= \frac{q(1-k^2) - 2k(1-k^q)}{(1-k)^2}
\end{aligned}
$$

- If $d := t - u \geq q - 1$

$$\begin{aligned}\operatorname{cov}(Y_t, Y_u) &= \sum_{i=1}^{q} \sum_{j=1+d}^{q+d} \mathbb{E}(X_i X_j) \\ &= \sum_{i=1}^{q} \sum_{j=1+d}^{q+d} k^{j-i} \\ &= \left(\frac{1-k^q}{1-k}\right)^2 k^{d+1-q}\end{aligned}$$

$$\operatorname{corr}(Y_i, Y_j) = \frac{(1-k^q)^2 k^{d+1-q}}{q(1-k^2) - 2k(1-k^q)} \tag{A5}$$

- If $d < q - 1$

$$\begin{aligned}\operatorname{cov}(Y_t, Y_u) &= \sum_{i=1}^{q} \sum_{j=1+d}^{q+d} \mathbb{E}(X_i X_j) \\ &= \frac{(q-d)(1-k^2) + k(k^{q+d} + k^{q-d} - 2k^d)}{(1-k)^2}\end{aligned}$$

$$\operatorname{corr}(Y_i, Y_j) = \frac{(q-d)(1-k^2) + k(k^{q+d} + k^{q-d} - 2k^d)}{q(1-k^2) - 2k(1-k^q)} \tag{A6}$$

□

Proof - Theorem 1. Using Lemma 1 and Lemma 2 on the sequence $(X_i^{(n)})_{1 \leq i \leq n}$ following an ARMA(1,q_n) with autocorrelation parameter $k^{1/(n-1)}$ and such that $q_n/n \overset{n\to\infty}{\longrightarrow} a$ (to check Assumption 2), we only have to take the limits of (A5) and (A6) for this particular sequence as $n \to \infty$.

$$\rho(x) = \lim_{n\to\infty, \frac{d}{n}\to x} \operatorname{corr}(Y_1, Y_{1+d})$$

□

Proof - Corollary 1.1. Taking $a = 0$ for an AR(1) process in Theorem 1 gives the correlation function.

In Lemma 1, the function $f(x, y, z)$ in the integral involves three terms, which simplify as follows in the case of the AR process with $0 < x < y < z < 1$:

$$r(0, x, y, z) = -\frac{\sqrt{1-k_{tot}^x}\sqrt{1-k_{tot}^{z-y}}k_{tot}^{y-x}}{2} \tag{A7}$$

$$r(0, y, x, z) = \frac{k_{tot}^{z-y} - k_{tot}^z - k_{tot}^{y-x} + k_{tot}^x}{2\sqrt{1-k_{tot}^y}\sqrt{1-k_{tot}^{z-x}}} \tag{A8}$$

$$r(0, z, x, y) = \frac{\sqrt{1-k_{tot}^{y-x}}(k_{tot}^{z-y} + k_{tot}^x)}{2\sqrt{1-k_{tot}^z}} \tag{A9}$$

Positivity for (A8)**:**

According to Lemma A1, $r(0,y,x,z)+r(0,z-y+x,x,z)\geq 0$, which implies that

$$2\int_0^1 (1-z)\int_0^z\int_0^y \arcsin(r(0,y,x,z))\mathrm{d}x\mathrm{d}y\mathrm{d}z \geq 0, \tag{A10}$$

using the fact that if $-1\leq a,b\leq 1$ and $(a+b\geq 0)$, then $(\arcsin(a)+\arcsin(b)\geq 0)$.
Positivity for (A7) + (A9)**:**

$$r(0,x,z-y+x,z)+r(0,z,x,y)=\frac{\sqrt{1-k_{tot}^{y-x}}\left(k_{tot}^{z-y}(1-\sqrt{1-k_{tot}^{x}}\sqrt{1-k_{tot}^{z}})+k_{tot}^{x}\right)}{2\sqrt{1-k_{tot}^{z}}}\geq 0$$

Finally, we use the fact that for $-1\leq a,b\leq 1, a+b\geq 0$,

$$\frac{\arcsin(a)+\arcsin(b)}{2}\geq \arcsin\frac{a+b}{2}.$$

□

Proof - Proposition 1. We conclude using (A4) and Lemma A3:

$$\begin{aligned}
\frac{\pi}{2}\binom{n-1}{2} &= \sum_{0<j<k<n}\left(L^n(j,k)+L^n(j,n-k+j)+L^n(n-k,n-k+j)\right) && \text{(Lemma } A3\text{)}\\
&= 3\sum_{0<j<k<n} L^n(j,k) && \text{(change of variable)}\\
\Rightarrow \frac{\pi}{6} &= \lim_{n\to\infty}\frac{1}{\binom{n-1}{2}}\sum_{0<j<k<n}\arcsin\left(\frac{k-j}{\sqrt{k}\sqrt{n-j}}\right)\\
\Leftrightarrow \frac{\pi}{6} &= \lim_{n\to\infty}\frac{1}{\binom{n+1}{4}}\sum_{0\leq i<j<k<l\leq n}\arcsin\left(\frac{k-j}{\sqrt{k-i}\sqrt{l-j}}\right) && \text{(using (A4))}
\end{aligned}$$

□

Proof - Corollary 1.2. Taking $k=0$ for a MA process in (A5) and (A6) gives the correlation function. Let $0<x<y<z<1$. As seen previously to prove Lemma 1:

$$\begin{aligned}
\mathbb{V}(\tau_n) &= \frac{2}{\pi\binom{n}{2}^2}\sum_{1\leq i<j\leq n}\sum_{1\leq k<l\leq n}\arcsin(\tilde{r}(i,j,k,l))\\
&= \frac{4}{\pi\binom{n}{2}^2}\Big(\sum_{1\leq i<j<k<l\leq n}\arcsin(\tilde{r}(i,j,k,l))+\sum_{1\leq i<k<j<l\leq n}\arcsin(\tilde{r}(i,j,k,l))\\
&\quad+\sum_{1\leq i<k<l<j\leq n}\arcsin(\tilde{r}(i,j,k,l))\Big)+o(1)
\end{aligned}$$

We consider two cases:

- If $a\geq 1$

For the first sum, we have that:

$$\forall n\geq 4, \sum_{1\leq i<j<k<l\leq n}\arcsin(\tilde{r}(i,j,k,l))=0,$$

Furthermore, for the second sum, from Proposition 1, 68

$$\lim_{n\to\infty}\frac{1}{\binom{n}{4}}\sum_{1\le i<k<j<l\le n}\arcsin(\tilde{r}(i,j,k,l))=\frac{\pi}{6}$$
$$\Leftrightarrow \lim_{n\to\infty}\frac{1}{\binom{n}{2}^2}\frac{2}{\pi}\sum_{1\le i<j\le n, 1\le k<l\le n}\arcsin\left(\tilde{r}(i,j,k,l)\right)=\frac{1}{9}$$

Finally, for the third sum, 69

$$r(0,z,x,y)=\sqrt{\frac{y-x}{z}},$$

hence: 70

$$\begin{aligned}\lim_{n\to\infty}\frac{4}{\pi\binom{n+1}{2}^2}\sum_{1\le i<k<l<j\le n}\arcsin(\tilde{r}(i,j,k,l)) &= \frac{16}{\pi}\int_0^1(1-z)\int_0^z\int_0^y\arcsin(\sqrt{\frac{y-x}{z}})\mathrm{d}x\mathrm{d}y\mathrm{d}z\\ &= \frac{16}{\pi}\int_0^1(1-z)\int_0^1\int_0^y z^2\arcsin(\sqrt{y-x})\mathrm{d}x\mathrm{d}y\mathrm{d}z\\ &= \frac{4}{3\pi}\int_0^1\int_0^y\arcsin(\sqrt{y-x})\mathrm{d}x\mathrm{d}y\end{aligned}$$

In order to calculate the last integral, we introduce λ between 0 and 1. 71

$$\sqrt{y-x}\le\lambda \iff y\le x+\lambda^2$$

Then the proportion of terms that verify this is equal to: 72

$$\begin{aligned}\frac{\int_0^1\int_x^1\mathbb{1}_{y\le x+\lambda^2}\mathrm{d}y\mathrm{d}x}{\int_0^1\int_x^1\mathrm{d}y\mathrm{d}x} &= 2\left(\int_0^{1-\lambda^2}\int_x^{x+\lambda^2}\mathrm{d}y\mathrm{d}x+\int_{1-\lambda^2}^1\int_x^1\mathrm{d}y\mathrm{d}x\right)\\ &= 2\lambda^2-\lambda^4\end{aligned}$$

The density is equal to $4\lambda(1-\lambda^2)$. 73

$$\int_0^1\int_x^1\arcsin\left(\sqrt{y-x}\right)\mathrm{d}y\mathrm{d}x=4\frac{\int_0^1\arcsin(\lambda)\lambda(1-\lambda^2)\mathrm{d}\lambda}{\int_0^1\int_x^1\mathrm{d}y\mathrm{d}x}$$

$$\begin{aligned}\int_0^1 t(1-t^2)\arcsin(t)\mathrm{d}t &= \left[\frac{t^2}{2}(1-\frac{t^2}{2})\arcsin(t)\right]_0^1-\int_0^1\frac{t^2}{2}(1-\frac{t^2}{2})\frac{1}{\sqrt{1-t^2}}\mathrm{d}t \text{ by IBP}\\ &= \frac{\pi}{8}-\int_0^{\pi/2}\frac{\sin^2(x)}{2}(1-\frac{\sin^2(x)}{2})\frac{\sqrt{1-\sin^2(x)}}{\sqrt{1-\sin^2(x)}}\mathrm{d}x\\ &= \frac{\pi}{8}-\frac{1}{2}\int_0^{\pi/2}\sin^2(x)\mathrm{d}x+\frac{1}{4}\int_0^{\pi/2}\sin^4(x)\mathrm{d}x\\ &= \frac{\pi}{8}-\frac{1}{4}\int_0^{\pi/2}\left(1-\cos(2x)\right)\mathrm{d}x+\frac{1}{16}\int_0^{\pi/2}\left(\frac{3}{2}-2\cos(2x)+\frac{1}{2}\cos(4x)\right)\\ &= \frac{3\pi}{64}\end{aligned}$$

Finally, we get, 74

$$\lim_{n\to\infty}\mathbb{V}(\tau_n)=\frac{1}{9}+2\frac{4}{3\pi}\frac{3\pi}{64}=\frac{17}{72} \tag{A11}$$

- If $0 < a < 1$

For $z < a$, the same formulas hold for r. Hence,

$$\frac{16}{\pi}\int_0^a (1-z)\int_0^z \int_0^y f(x,y,z)\mathrm{d}x\mathrm{d}y\mathrm{d}z = \frac{17}{72}\frac{\int_0^a (1-z)z^2\mathrm{d}z}{\int_0^1 (1-z)z^2\mathrm{d}z} \tag{A12}$$

$$= \frac{17}{72}(4a^3 - 3a^4) \tag{A13}$$

For $z > a$, one can check that:

$$r(0,y,x,z) + r(0,z-y+x,x,z) \geq 0$$

$$r(0,x,z-y+x,z) + r(0,z,x,y) \geq 0$$

□
