# Supplementary material for: On the Gaussian distribution of the Mann-Kendall tau in the case of autocorrelated data
Source: PLoS One. 2026 Feb 19;21(2):e0333224. doi: 10.1371/journal.pone.0333224 (PMC12919832; doi:10.1371/journal.pone.0333224)
Supplement: Attachment — Submitted filename: Report on On the Gaussian distribution ....pdf [file pone.0333224.s002.pdf]

Report on

“Conditions Under Which the Kenward-Roger Approximate Test is an Exact”

The manuscript explores an asymptotic framework for autoregressive processes of order 1 and simple moving 12average processes of order q. The authors prove that, along with upsampling sequences, the distribution of the normalized Mann-Kendall tau does not converge to a Gaussian.

On the whole, the subject of this manuscript is interesting. The statistical model and the equations are derived well, and the numerical results are adequate. However, the manuscript needs minor corrections. The following points need to be corrected:

1- All symbols need to be defined, for example $E(A_{ii}, A_{kl})$ and $\binom{n}{2}^2$ in equation 3 and $\lfloor an \rfloor$ in example 2.

2- The outline of “Section 3”, “Section 4” is repeated on pages 2 and 3.

3- On page 4, the ϵ_i^n should be $\epsilon_i^{(n)}$

4- The figures should be between the text where they are mentioned.
