# Supplementary material for: On the Gaussian distribution of the Mann-Kendall tau in the case of autocorrelated data
Source: PLoS One. 2026 Feb 19;21(2):e0333224. doi: 10.1371/journal.pone.0333224 (PMC12919832; doi:10.1371/journal.pone.0333224)
Supplement: Attachment — Submitted filename: Response to Reviewers.pdf [file pone.0333224.s003.pdf]

Dear Editor,

Thank you for your generally positive evaluation. We have addressed the report and the corrections requested by you and the two reviewers. You will find in the rest of this document responses to each point that was raised.

We noticed that there was an error in the header of the report of the first reviewer, which did not indicate the correct article title, although the reviewer's comments do refer to our manuscript.

Best regards,
The authors

## Regarding the journal requirements

1. Please ensure that your manuscript meets PLOS ONE's style requirements, including those for file naming. [...]

→ We have taken care to ensure that these requirements are met.

2. Thank you for stating the following financial disclosure: [AAP2023 FIRE mini-grant]. Please state what role the funders took in the study. [...]

→ The funders had no role in study design, data collection and analysis, decision to publish, or preparation of the manuscript.

3. When completing the data availability statement of the submission form, you indicated that you will make your data available on acceptance. We strongly recommend all authors decide on a data sharing plan before acceptance, as the process can be lengthy and hold up publication timelines. [...]

→ We have uploaded our code files as Supporting Information during the Plos One submission process. We plan on making these files freely accessible for the readers, either through files in the Supporting Information Section, or through the following github: https://github.com/Nils-TS/On_the_Gaussian
We are fine with one or both options; and will follow your recommendation. In any case, the code files are available on the github page.

4. We are unable to open your Supporting Information/other file [Code_Figures.ipynb]. Please kindly revise as necessary and re-upload.

→ We were surprised that you were unable to open this document, which is a standard Python Jupyter Notebook file. Most Python environments allow you to open and execute this type of code. A preview of the document is also accessible at https://github.com/Nils-TS/On_the_Gaussian/blob/main/Figures.ipynb (github with our freely accessible code) which would alternatively allow you to copy and paste the different pieces of Python code present in the Jupyter Notebook file.

## Regarding the reviewers comments to the authors

*In the following, line numbers refer to the revised version of the manuscript with highlighted changes made to the original version.*

### Reviewer #1:

On the whole, the subject of this manuscript is interesting. The statistical model and the equations are derived well, and the numerical results are adequate.

→ *Thank you for this appreciation.*

1- All symbols need to be defined [...]

→ *These definitions have been added, see L70, L56, example four, as well as L63 for the variance of the random variable.*

2- The outline of “Section 3”, “Section 4” is repeated on pages 2 and 3.

→ *redundancies have been removed. We removed L84-87 "Section 3 then explores cases in which this assumption holds, specifically for time series generated by AR(1) and SMA processes. Finally, section 4 numerically illustrates the practical consequences which this result has on the classic Mann-Kendall test for trend testing."*

3- On page 4, the $\epsilon_i^n$ should be $\epsilon_i^{(n)}$

→ *This is corrected, see L98 with example one.*

4- The figures should be between the text where they are mentioned

→ *We respected the Plos One submitting template for our revised manuscript, which requires that they are uploaded separately as individual files. However, each figure caption appears directly after the paragraph in which it is first cited.*

### Reviewer #2:

I commend the authors for choosing a vital theme in longitudinal analyses: that of modifying asymptotic guarantees to finite-sample contexts and I like their organization, analysis and presentation.

→ *Thank you for this appreciation of our work.*

Is it possible to upgrade one step higher on your first example: from an autoregressive to a threshold autoregressive (TAR) process? In particular, if the finiteness doesn’t impact the pre-threshold phase and the post-threshold phase equally? How would the scaling alter? Maybe some simulations here?

*→ Thank you for this suggestion to extend the scope of our study. We have used your remark to extend our discussion.*

*Threshold autoregressive models (Tong 1978) are non-linear time series models which can be seen as mixture models of autoregressions for which thresholds on an instrumental variable behaving as a concomitant variable (Grun and Leisch 2008) decide which component autoregression applies at each specific time point in the series. Tsay (1989) proposed self-exciting TAR (SETAR) as a non-linear time series model where the instrumental variable is the response itself, showing a wider range of stationary behaviour than linear autoregressions (they can model for example so called one-hump maps). The focus for these models seems to have been on identification of parameters. Our analysis of stationary distributions would imply that a TAR or SETAR extension of our results would start from cases where the process does not have thresholds and instrumental variables which divide the time series of the response in clear time phases (no trends, null model). Anděl et al. (1984) investigated some specific SETAR and derive their stationary distributions analytically for the simplest case and numerically for others. It is our opinion that TAR are to be considered non-linear time series models, and that it seems more naturally to study their distributions and asymptotic variances in the absence of trends purely numerically, which is a different approach than what we investigated here. We therefore cite a reference on TAR and SETAR processes along other methods of forecasting and trend detection than Mann-Kendall's tau distributions expected from AR(1) and MAR processes in the paragraph where we broaden our discussion. We are preparing a second separate paper on the distributions of Mann-Kendall's tau in non-linear models.*

*References not cited in main revised text:*
*Anděl J et al (1984) On threshold autoregressive processes. Kybernetika 20: 89-106*
*Grun, B. & Leisch, F. (2008). FlexMix version 2: finite mixtures with concomitant variables and varying and constant parameters. Journal of Statistical Software, 28 (4), 1-35.*
*Tong, H. (1978) On a Threshold Model in Pattern Recognition and Signal Processing. In: Chen, C., Ed., Pattern Recognition and Signal Processing, Sijhoff and Noordhoff, Amsterdam, 575-586.*

I'd encourage the authors to enlarge the discussions a bit more.

*→ Thank you for this remark. Most of our changes are made in the discussion. Please inspect the last paragraph of the discussion which integrates several of your remarks.*

For instance, I'd be curious to know the impacts these may have on detecting changes in trend and how would those compare with other recent techniques such as e-divergence or prophet-type strategies. A mention or acknowledgement of these would go a long way even if the authors do not deploy a large-scale analysis to examine these things on the current paper.

*→ We mention and acknowledge the existence of different types of trend detection methods. such as the prophet-type models. This allowed us to broaden our discussion.*

Maybe motivate the analyses (or mention in the discussions) with some non-time series anecdotes/contexts where the departure (from theoretical asymptotics) due to finiteness had a crucial role to play in policy, people's lives, etc.

*→ We have added such context, regarding broad ongoing discussions on asymptotic results and Monte-Carlo exact methods for finite samples, on L28-34 in the introduction. Our results (Figures one and two) indicate that our asymptotic results remain relevant for time series of short lengths (a few tens of data points), when the individual errors are Gaussian. We stress this in the discussion on L418-420.*

I like the approach the authors have taken, especially in defining/stressing repeatedly what they are trying to achieve (point out a deviation from normality), but we can be mindful of Plos One's diverse readership. If in a small fresh section, the authors can add small numerical examples to clarify structures that may seem technical to some (sequence of sequences, etc.), that could ensure wider accessibility with the article being self-contained (i.e., without the readers having to visit references (hydrological, for instance) the authors have cited). If this exceeds length requirements, please ignore.

*→ We have added an example at the first occurrence of the sequence of sequences, in Example 1 on L102-112.*

Do the authors plan on developing software packages (for example, an R package) where someone can enter a finite-length time series and out will come a decision (through their Shapiro-Wilks choice) that says whether normality is okay and/or output the right scaling in case it is not? They may mention this as a future possibility as well.

*→ An R package would be overkill at this point for a relatively simple decision, but remains an option in combination with different related results. As a supplement, we have added functions for R and Python which carry out the calculation of the criterion for autoregressive models (accessible via the following github repository that will be available in the data availability Section* https://github.com/Nils-TS/On_the_Gaussian *as explained on L428-429). For SMA processes, the result requires no calculation, namely that the moving window should be below 10% of the series length. Alternatively, when it wouldn't be known which relative size the windows were, an estimation procedure is proposed, but its properties haven't been investigated yet, see L435-440.*

Some minor concerns that another round of edits could allay:
For example, on such lines as 34-36, page2: "Concerning the SMA(q) process, these time series amount to averaging a larger and larger sample of a white noise process with a constant relative
window size, which can be used as the null model when testing for the presence of critical transitions in time series - also known as early warning signals [8]."
- "Regarding" instead of "concerning"? Did you mean a "larger and larger sample" or a "larger sample". If the former, i.e., a sequential change-point-type approach, please consider being more explicit.

*→ We addressed these concerns, please inspect L38-39, L202, L430.*
